# Discrete terminal ileal ulcers in patients diagnosed with ulcerative colitis: clinical significance and natural course

**DOI:** 10.1186/s12876-021-01866-7

**Published:** 2021-07-12

**Authors:** Hyo-Jin Lim, Hyun Do Kim, Jae Seung Soh, Sung-Yeun Kim, Ye-Ji Jung, Hyun Lim, Ho Suk Kang, Jong Hyeok Kim

**Affiliations:** Department of Internal Medicine, University of Hallym College of Medicine, Hallym University Sacred Heart Hospital, 22 Gwanpyeong-ro 170-gil, Dongan-gu, Anyang, 14068 Republic of Korea

**Keywords:** Terminal ileum, Ulceration, Ulcerative colitis, Colonoscopy

## Abstract

**Background:**

Terminal ileal (TI) ulcers are occasionally detected in asymptomatic individuals and mostly resolve without any treatment. In patients with ulcerative colitis (UC), TI ulcers are infrequently observed without evidence of backwash ileitis. However, the clinical significance and natural course of the lesions are unclear. The aim of our study was to evaluate the frequency and clinical implications of TI ulcers in patients with UC.

**Methods:**

We retrospectively reviewed 397 patients with UC via successful TI intubation during colonoscopy. We compared the clinical characteristics of patients manifesting TI ulcers with those who did not. The natural course of TI lesions was also investigated during the follow-up periods.

**Results:**

Forty-one patients (10.3%) showed TI ulcers without evidence of inflammation in the right colon. The patients with and without TI ulcers were not different in terms of baseline characteristics, disease activity and extent at the time of the UC diagnosis, proximal extension, Mayo endoscopic score at the last endoscopic examination, medication history, UC-related hospitalization, and relapse during follow-up periods. Of the 30 patients who underwent follow-up colonoscopy in patients with TI ulcers, 23 (76.7%) showed resolution of TI ulcer. In addition, patients with remaining TI ulcers did not differ in disease activity and biopsy results compared with those with resolving TI ulcers.

**Conclusions:**

Discrete TI ulcers are more common in patients with UC, compared with the healthy cohort. No significant clinical impact on disease extension and severity is found.

## Introduction

Discrete terminal ileal (TI) ulcers are detected occasionally in asymptomatic healthy individuals regardless of medication history including treatment with aspirin or nonsteroidal anti-inflammatory drugs (NSAIDs). Most lesions detected in the terminal ileum resolve completely spontaneously without treatment, while a few TI ulcers are associated with significant diseases such as intestinal tuberculosis or Crohn’s disease [1]. Most asymptomatic individuals with TI ulcers show a benign natural course, which does not require empirical therapy.

Patients treated for ulcerative colitis (UC) also carry infrequent aphthous or small ulcerations in terminal ileum during ileocolonoscopy, which differ from backwash ileitis that shows inflammatory reaction in distal ileum following reflux of colonic contents, induced by malfunction of ileocecal (IC) valve [2, 3]. These cases of UC frequently manifest disease extension involving the whole colon including cecum and IC valve associated with severe disease activity. However, TI ulcers detected in cases not involving cecum or ascending colon can be explained via another mechanism besides backwash ileitis [4]. In addition, clinical significance and natural course of discrete TI ulcers in patients with UC were not fully evaluated.

This study aimed to evaluate the frequency of discrete TI ulcers in patients with UC and determine the clinical significance and the natural course of UC in patients carrying TI ulcers compared with those who do not.

## Methods

### Study patients

We retrospectively analyzed the medical and endoscopic records of patients diagnosed with UC at the Hallym University Sacred Heart Hospital in Anyang, Korea between April 2000 and December 2020. The colonoscopic examination was performed in 485 patients with UC at least once during this period. Eighty-eight of these patients were excluded due to sigmoidoscopic examination alone (44 patients), unsuccessful ileal intubation (25 patients), and unclear or changed diagnosis to Crohn’s disease or intestinal tuberculosis (19 patients). We evaluated the clinical information of the remaining 397 patients (e.g., sex, date of birth, date of UC diagnosis, smoking history, family history, extraintestinal manifestation, disease extent and activity, history of treatment with corticosteroids, azathioprine and/or 6-mercaptopurine, and anti-TNF agents, history of colectomy or UC-related hospitalization and relapse during follow-up periods). Disease extent was categorized as proctitis (rectum only), left-sided (rectum to descending colon), and extensive (rectum to more than transverse colon). Disease activity was graded as inactive (score 0–2), mild (3–5), moderate (6–10), or severe (11–12) based on the Mayo score (4 categories each scoring 0–3 for stool frequency, rectal bleeding, findings at colonoscopy, and physician global assessment) [5]. The Mayo endoscopic score (MES) was classified into the following four categories: 0, normal or inactive disease; 1, mild disease with erythema, decreased vascular patterns and mild friability; 2, moderate disease with marked erythema, absence of vascular patterns, friability and erosions; 3, severe disease with spontaneous bleeding and ulceration. Maximal disease extent was defined as maximum degree of colonic inflammation to investigate proximal disease extension during the follow-up periods. UC-related hospitalization was defined as admission at our hospital because of disease aggravation or adverse events related to medication. Relapse was defined by the use of corticosteroids or other medications because of disease aggravation clinically or endoscopically. The study protocol was approved by the Institutional Review Board of Hallym University Sacred Heart Hospital, Anyang, Korea (No. 2020-12-006-002). All methods were carried out in accordance with relevant guidelines and regulations.

### Study colonoscopy

All colonoscopies were performed by staff gastroenterologists and their fellows at the Hallym University Sacred Heart Hospital using standard colonoscopes. TI ulcers were defined as definite mucosal breaks in the terminal ileum, and not simple petechiae or hyperemic lesions. We excluded patients with continuous inflammatory reactions up to cecum and ascending colon or patulous IC valve. However, patients with inflammation involving only the appendiceal orifice were enrolled. More than 80% patients had undergone biopsies of the TI ulcers, and histology was evaluated by gastrointestinal pathologists who were skilled at histological analysis of inflammatory bowel disease (IBD). We assessed the follow-up colonoscopies of patients with TI ulcers to identify whether or not the lesions remained or resolved.

### Statistical analyses

The chi-square test or 2-tailed Fisher’s exact test was used to evaluate the association between various categorical variables, and independent sample *t *test was used for non-categorical variables. The Statistical Package for the Social Sciences (SPSS) version 22.0 (SPSS Inc., Chicago, IL, USA) was used for all statistical analyses. A *P *value of < 0.05 was considered statistically significant.

## Results

### Prevalence of patients with TI ulcers in UC

Of the 397 UC patients enrolled in our study, 41 patients showed discrete TI ulcers during ileocolonoscopy. The prevalence of TI ulcers was 10.3%. At the time of identifying TI ulcers, two patients were taking 100 mg of aspirin for hypertension and one patient was taking NSAID (400 mg of ibuprofen) for pharyngitis. Other patients were not taking any medication including aspirin, NSAID, or antibiotics, which might cause discrete TI ulcers.

### Comparison of clinical features between patients with and without TI ulcers

We allocated the 41 patients with TI ulcers to group A and the remaining 356 patients to group B. There was no significant difference in baseline characteristics including age, age at the time of UC diagnosis, sex, smoking and family history, and the prevalence of extraintestinal manifestations (e.g., arthritis, stomatitis, ankylosing spondylitis, psoriasis, uveitis, erythema nodosum, primary sclerosing cholangitis, and epididymitis) between the groups A and B (Table 1). Inflammation of the appendiceal orifice without inflammation of the ascending colon did not differ between the two groups. Disease activity and extent at the diagnosis of UC showed no significant differences between groups A and B (*P* = 0.173 and 0.472, respectively). Maximal extent and proximal extension during follow-up periods were also not different. Five patients showed proximal extension in group A (12.2%) compared to 69 patients in group B (16.9%) (*P* = 0.396). About 70% of patients were showed 0 to 1 Mayo endoscopic score at the last colonoscopic examinations in both groups. In addition, the history of medication use including corticosteroids, azathioprine/6-mercaptopurine, cyclosporine, and anti-tumor necrotizing factor was not significantly different. UC-related hospitalization and relapse during follow-up periods were also not different between the two groups (*P* = 0.719 and 0.502, respectively).

**Table 1:** Clinical manifestations of UC patients with and without TI ulcers

Variables	Group A	Group B	*p* value
	(n = 41)	(n = 356)	
Age, years, mean (SD)	48.3 (15.3)	48.5 (16.7)	0.909
Age at the diagnosis of UC, years, mean (SD)	39.8 (15.5)	40.0 (15.7)	0.929
Male sex, no. (%)	25 (61.0)	221 (62.1)	1.000
Smoking history, no. (%)			0.891
Never smoker, no. (%)	25 (61.0)	212 (59.6)	
Former smoker, no. (%)	11 (26.8)	111 (31.2)	
Current smoker, no. (%)	5 (13.2)	33 (9.3)	
Family history, no. (%)	1 (2.4)	5 (1.4)	0.482
Extraintestinal manifestations, no. (%)	2 (4.9)	47 (13.2)	0.205
Inflammation of the appendix orifice, no. (%)	10 (24.4)	89 (25.0)	1.000
Disease activity at the diagnosis of UC			0.851
Mild/inactive, no (%)	23 (56.1)	199 (55.9)	
Moderate, no. (%)	14 (34.1)	130 (36.5)	
Severe, no. (%)	4 (9.8)	27 (7.6)	
Disease extent at the diagnosis of UC			0.173
Proctitis, no. (%)	18 (43.9)	193 (54.2)	
Left-sided colitis, no. (%)	11 (26.8)	87 (24.4)	
Extensive colitis, no. (%)	12 (29.3)	76 (21.3)	
Maximal extent, no. (%)			0.472
Proctitis, no. (%)	13 (31.7)	142 (39.9)	
Left-sided colitis, no. (%)	14 (34.1)	99 (27.8)	
Extensive colitis, no. (%)	14 (34.1)	115 (32.3)	
Proximal extension, no. (%)	5 (12.2)	69 (19.4)	0.396
Mayo endoscopic score at the last endoscopy			1.000
0–1, no. (%)	29 (70.7)	253 (71.1)	
2–3, no. (%)	12 (29.3)	103 (28.9)	
Ever use of medications			
Corticosteroid, no. (%)	23 (56.1)	197 (55.3)	1.000
Azathioprine/6-MP, no. (%)	16 (39.0)	108 (30.3)	0.286
Anti-TNF agents, no. (%)	3 (7.3)	19 (5.3)	0.486
Colectomy, no. (%)	1 (2.4)	1 (0.3)	0.196
UC-related hospitalization, no. (%)	13 (31.7)	103 (28.9)	0.719
Relapse during follow-up periods, no. (%)	19 (46.3)	142 (39.9)	0.502
Follow-up duration, months, mean (SD)	78.1 (60.4)	70.1 (59.9)	0.415

TI, terminal ileum; SD, standard deviation; UC, ulcerative colitis; MP, mercaptopurine; TNF, tumor necrosis factor

### Ileocolonoscopic and histologic features of TI ulcers

**Fig. 1:**
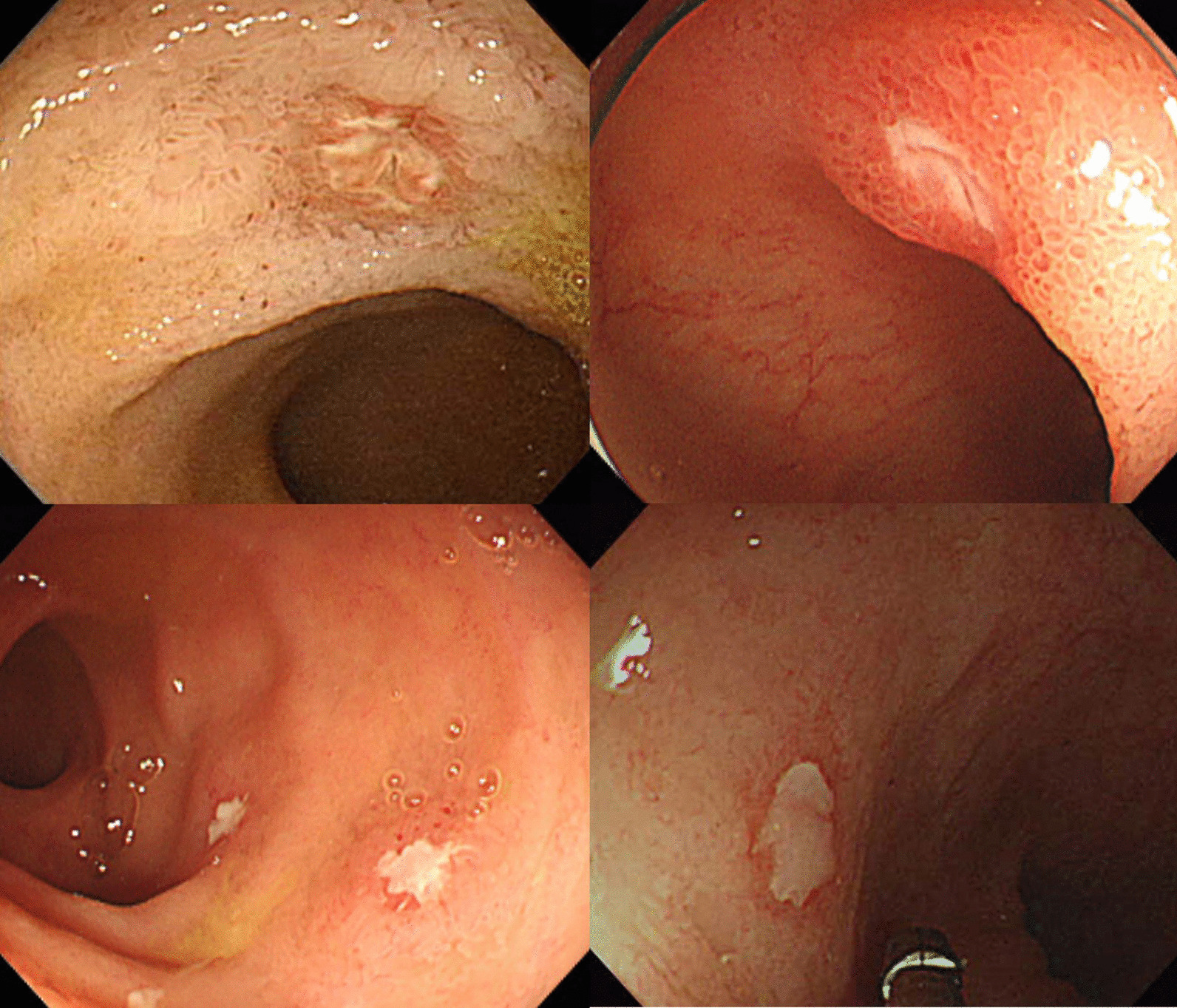
Colonoscopic images of terminal ileal ulcers in patients with ulcerative colitis

Figure 1 shows ileocolonoscopic images of TI ulcers in UC patients. Most of the ulcerations were surrounded by normal or edematous mucosa, and/or regenerating epithelium, and covered with exudate. Twenty-five patients (61.0%) carried single ulcers and the rest of the patients showed more than two ulcerations. The maximal size of ulcers was estimated to be < 5 mm in 31 patients (75.6%), and ≥ 5 mm in 10 (24.4%). Ten patients (24.4%) showed inflammation involving the appendiceal orifice in conjunction with terminal ileum. At the time of detecting TI ulcers, 26 patients (63.4%) presented inactive or mild disease activity and the remaining 15 patients (36.6%) had moderate activity. Among the 12 patients with extensive disease at the time of the UC diagnosis, four showed remission and the remaining eight showed disease extension below transverse colon at the time of detecting TI ulcers.

**Fig. 2:**
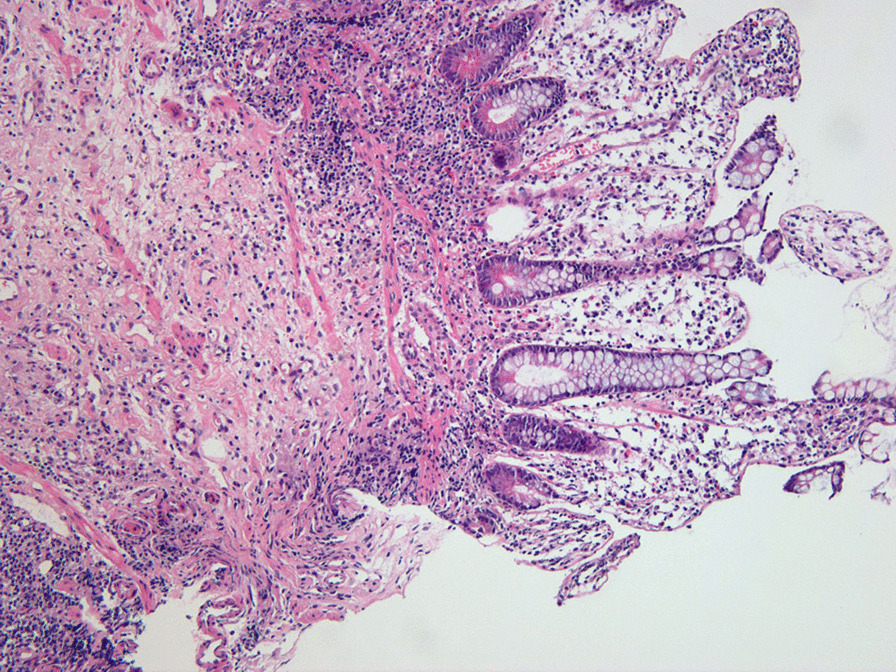
Histopathological image of discrete terminal ileal ulcer in patients with ulcerative colitis

Biopsies of 34 patients were obtained including 30 patients with TI ulcers (88.2%) pathologically diagnosed with chronic or nonspecific inflammation with or without lymphoid hyperplasia. The lesions of the remaining four patients were diagnosed as active or erosive ulcerations. Figure 2 shows a representative histopathological image of TI ulcer that left side of the lesion was observed submucosal fibrosis by ulceration and right side of the lesion was showed a minimal erosion on villous epithelium.

### Natural courses of terminal ileal ulcers

**Fig. 3:**
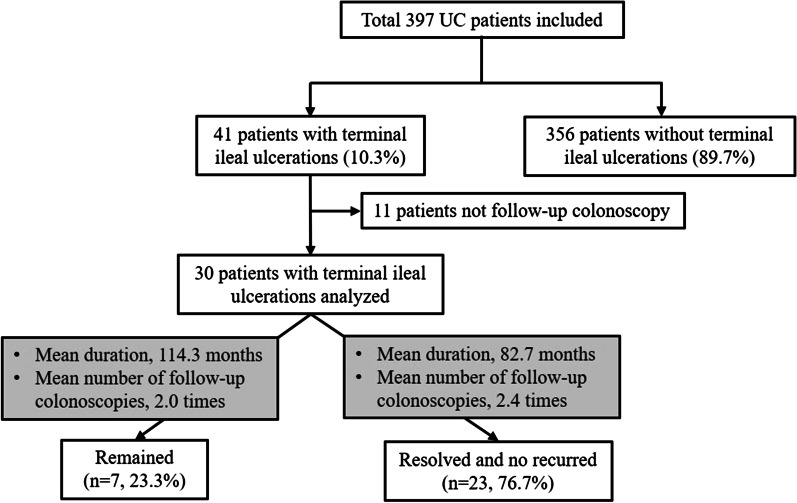
Flowchart of patients recruited from the study population and the natural course during the follow-up periods

Figure 3 shows the clinical courses, the follow-up periods, and the mean number of follow-up colonoscopies of enrolled patients. The excluded 11 patients did not undergo follow-up colonoscopy, whereas seven (23.3%) of 30 patients showed continuing TI ulcers during the follow-up colonoscopy. The mean follow-up duration of these patients was 114.3 months (range 32–233 months) and the mean number of follow-up colonoscopies was 2.0 times (range 1–5). Three of the lesions showed no significant changes or partial improvements compared with initial colonoscopy, whereas three showed waxing and waning, and one showed aggravation. However, 23 (76.7%) TI ulcers were completely resolved without recurrent during the follow-up colonoscopy. The mean follow-up duration was 82.7 months (range 14–234 months) and the mean number of follow-up colonoscopies was 2.4 times (range 1–8). In these patients, the mean duration until initial resolution of TI ulcers was 26.0 months (range 4–59 months) and the mean number of colonoscopies was 1.2 times (range 1–3). Thirty patients underwent esophagogastroduodenoscopy during the follow-up periods. Three patents were diagnosed with gastric ulcer and one patient was duodenal ulcer. Of these four patients, one patient was included in the group of continuing TI ulcer.

## Discussion

In this study, we found that TI ulcers were more prevalent in patients with UC than in asymptomatic individuals. However, clinical implications including disease extent and activity of UC patients with TI ulcers did not differ compared with those of patients with UC without TI ulcers. During the follow-up periods, three-fourths of TI ulcers were resolved. TI lesions of UC patients showed benign natural courses similar to those of asymptomatic individuals.

A few studies evaluating the diagnostic results of ileocolonoscopy also investigated the TI inflammatory lesions in the terminal ileum. A study investigating TI ulcers in asymptomatic individuals without gastrointestinal symptoms or history of NSAID use at the department of Health Promotion Center showed a prevalence rate of 0.4% (106 of 26,735), while our study showed a prevalence of 10.3% in patients with UC [1]. Another study evaluating the diagnostic value of terminal ileum intubation in Korea showed that 83 (2.4%) of 3,417 cases carried ulcers or erosions during routine ileocolonoscopy [6]. The abnormality rate (2.4%) of terminal ileum in this study was higher than 0.4% in the healthy cohort, which was attributed to the inclusion of patients manifesting symptoms including abdominal pain or diarrhea in the Department of Gastroenterology, but not in the Department of Health Promotion Center. Previous studies from the West showed that the prevalence of terminal ileal abnormalities was 1.9–4.6% [7–9]. However, these studies included terminal ileal abnormalities such as mucosal nodularity or erythema, and polypoid lesions as well as ulcers. Patients with UC in the current study manifested mostly symptoms including diarrhea, mucoid or bloody stools, and abdominal pain; therefore, abnormalities of terminal ileum might be more prevalent compared with studies involving general patients or healthy cohorts. Hamilton et al*.* showed the rate of ileitis in UC patients was higher than in non-UC control (22% vs. 4%, *P* < 0.001), although the number of enrolled patients was limited (72 patients for UC and 90 patients for control) [10].

Hamilton et al*.* also compared patients with UC along with ileitis (n = 16) and patients without ileitis (n = 56). There was no significant difference in the clinical severity scores and the overall extent of colon involvement between the two groups. Our study showed similar results not only in disease activity and maximal disease extent, but also in proximal extensions, UC-related hospitalization, and relapse during follow-up periods, even though our study included a higher number of patients with UC (n = 397). In addition, we investigated history of medications including corticosteroids, azathioprine/6-mercaptopurine, and anti-tumor necrotizing factors during the follow-up period to evaluate disease severity. The history of medication use was not significantly different between patients with and without TI ulcers. TI ulcers detected in patients with UC were considered not to influence the disease course.

A study of asymptomatic individuals showed that TI ulcers were resolved in two-thirds of 93 patients, while persisting in one-third of patients [1]. Two patients received anti-tuberculosis treatment in the former group and one patient progressed to Crohn’s disease in the latter group. The natural course of patients with UC did not differ from asymptomatic individuals. The lesions of 77% of patients with TI ulcers were found to have resolved during the mean follow-up period of 6.8 years. The results of all seven patients with persistent TI ulcers during the follow-up did not differ and showed no exacerbation compared with 23 patients with resolved TI ulcers. Pathologic evaluation of TI ulcer is crucial to exclude specific diseases such as lymphoma, Crohn’s disease, or tuberculosis [11–13]. However, TI ulcers diagnosed pathologically with chronic or nonspecific inflammation showed insignificant clinical features in patients with UC.

Ileal inflammation in patients with UC has been attributed to the reflux of colonic contents into the ileum because of incompetent and inflamed ileocecal valve, which was described as backwash ileitis [14]. To prove this theory, patients should exhibit severe activity and colonic involvement, especially in the cecum and ascending colon. In addition, similar histological findings of ileum and colon are necessary [2]. Other pathogenetic mechanisms should be considered in case of ulcers of the terminal ileum without patulous ileocecal valve or inflammation. Discrete TI ulcers might be associated with UC-related medication, NSAIDs, smoking, or alcohol. However, the lesions may present as extracolonic manifestations of UC, similar to the involvement of the upper gastrointestinal tract [2, 4, 10]. The precise etiology of TI ulcers is unknown. Further studies under strict and uniform pathologic criteria are needed to demonstrate clinical associations.

The current study has several limitations. First, the retrospective and non-randomized analysis conducted at a single center could have resulted in biases due to unrecognized or unmeasured factors. Second, our study did not include a control group, which was not diagnosed with UC. The incidence of TI ulcers could not be comparable without control group. Third, some of TI ulcers in our study could be caused by backwash ileitis. Ten patients (24.4%) showed inflammation on the appendiceal orifice and 12 patients had extensive colitis at the time of UC diagnosis. Although the patients were in remission or manifested disease extent below the transverse colon, the effect on previous inflammation in the ascending colon and cecum could not be excluded. Hamilton et al*.* selected patients with UC manifesting only inactive or mild disease activity to exclude backwash ileitis [10]. Twenty-six patients (63.4%) in our study showed inactive or mild disease activity at the time of TI ulcers and none manifested severe disease activity. Lastly, a few patients were taking aspirin or NSAID in the group with TI ulcers. In addition, the information of bowel preparation was limited in the present study. NSAIDs and agents for bowel cleansing might contribute to TI ulcers.

In conclusion, discrete TI ulcers are considered to be more common in UC patients, compared with previous studies of non-UC patients. Three-fourths of the lesions were resolved without any treatment. Clinical impact on disease extension and severity appears insignificant during follow-up. A further multi-center study including a large cohort is needed to corroborate the results.

## Data Availability

The datasets used and/or analyzed during the current study are available from the corresponding author on reasonable request.
